# Local climate zone-based urban land cover classification from multi-seasonal Sentinel-2 images with a recurrent residual network

**DOI:** 10.1016/j.isprsjprs.2019.05.004

**Published:** 2019-08

**Authors:** Chunping Qiu, Lichao Mou, Michael Schmitt, Xiao Xiang Zhu

**Affiliations:** a Signal Processing in Earth Observation (SiPEO), Technical University of Munich (TUM), Arcisstr. 21, 80333 Munich, Germany; b Remote Sensing Technology Institute (IMF), German Aerospace Center (DLR), Oberpfaffenhofen, 82234 Wessling, Germany

**Keywords:** Land cover, Local climate zones (LCZs), Sentinel-2, Multi-seasonal, Residual convolutional neural network (ResNet), Long short-term memory (LSTM), Recurrent neural network (RNN)

## Abstract

The local climate zone (LCZ) scheme was originally proposed to provide an interdisciplinary taxonomy for urban heat island (UHI) studies. In recent years, the scheme has also become a starting point for the development of higher-level products, as the LCZ classes can help provide a generalized understanding of urban structures and land uses. LCZ mapping can therefore theoretically aid in fostering a better understanding of spatio-temporal dynamics of cities on a global scale. However, reliable LCZ maps are not yet available globally. As a first step toward automatic LCZ mapping, this work focuses on LCZ-derived land cover classification, using multi-seasonal Sentinel-2 images. We propose a recurrent residual network (Re-ResNet) architecture that is capable of learning a joint spectral-spatial-temporal feature representation within a unitized framework. To this end, a residual convolutional neural network (ResNet) and a recurrent neural network (RNN) are combined into one end-to-end architecture. The ResNet is able to learn rich spectral-spatial feature representations from single-seasonal imagery, while the RNN can effectively analyze temporal dependencies of multi-seasonal imagery. Cross validations were carried out on a diverse dataset covering seven distinct European cities, and a quantitative analysis of the experimental results revealed that the combined use of the multi-temporal information and Re-ResNet results in an improvement of approximately 7 percent points in overall accuracy. The proposed framework has the potential to produce consistent-quality urban land cover and LCZ maps on a large scale, to support scientific progress in fields such as urban geography and urban climatology.

## 1 Introduction

**Fig. 1:**
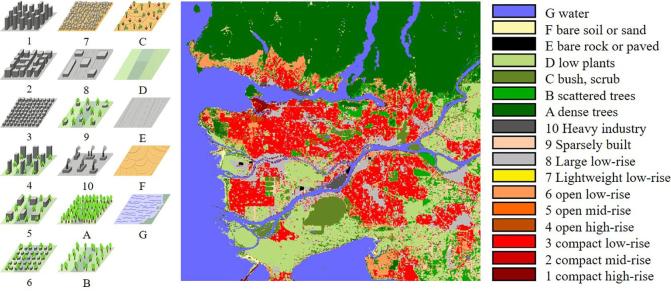
LCZ definition and an sample LCZ map of Vancouver, Canada. The left subfigure was modified from WUDAPT (Stewart, 2011).

The local climate zone (LCZ) scheme has been developed primarily for the communication of meta-data produced by observational urban heat island (UHI) studies and has a broad range of applications, including classifying weather stations and assessing social inequality (Stewart, 2011). The 17 LCZs that have been developed document climate-related surface properties at a local scale in urban environments. The LCZ scheme primarily considers surface cover (e.g., paved, low plants, or water), 3D surface structure (e.g., height and density of buildings and trees), and anthropogenic parameters such as anthropogenic heat output from human activity (Demuzere et al., 2019). The scheme classifies urban landscapes into ten “built” and seven “natural” zones, as shown in Fig. 1. It is intended to be universally applicable to urban environments worldwide, thereby offering the possibility of comparative analysis and the monitoring of portions of different cities across the world in a consistent manner (Bechtel et al., 2015).

In addition to its strong usefulness in urban climate studies (Stewart and Oke, 2012, Stewart et al., 2014, Fenner et al., 2017, Quan et al., 2017, Quanz et al., 2018, Kotharkar and Bagade, 2018), the potential of LCZ for classifying the internal urban structure of human settlements, to provide auxiliary data for applications such as disaster mitigation, urban planning, and population assessment (Bechtel et al., 2016, Wicki and Parlow, 2017) in a rapidly urbanizing world (Taubenböck et al., 2012) has recently been explored. Furthermore, accurate LCZ maps can be used to extract and analyze reliable and detailed information on the extent of human settlement to provide assistance in fulfilling the evaluation and monitoring requirements of the 2030 Agenda for Sustainable Development and provide reference information for achieving the Sustainable Development Goal 11 (United and Nations, 2015), “Make cities and human settlements inclusive, safe, resilient, and sustainable.” As an example of such applications, the LCZ framework was exploited to monitor sustainable urbanization in terms of access to safe housing using data from an exemplary study in Pretoria and Johannesburg, South Africa (Danylo et al., 2017).

Current large-scale LCZ mapping efforts are generally community-based projects that use freely available Landsat data and free software packages (Mills et al., 2015, Bechtel et al., 2015, Ren et al., 2016). The overall accuracies (OAs) of such classification results range from 60 to 90% and depend primarily on the knowledge and capabilities of volunteers (Ren et al., 2016, Bechtel et al., 2017, Demuzere et al., 2019). Furthermore, community-based approaches are not well-suited to achieving large-scale mapping that needs to be frequently updated or to monitoring changes at fine temporal resolutions.

Another promising approach is supervised automatic LCZ classification with satellite remote sensing images (Yokoya et al., 2017, Qiu et al., 2018, Zhang et al., in press, Hu et al., 2018), which can potentially be used for LCZ mapping on a worldwide scale without relying on local expert knowledge for each individual city (Mills et al., 2015). However, large-scale LCZ mapping remains difficult because of the unavailability of sufficient high-quality training data, which hinders the generalization of trained classifiers. This challenge has roots in the large intra-class variability of spectral signatures resulting from regional variations in artificial materials and vegetation and other variations in physical and cultural environmental characteristics (Bechtel et al., 2015). Related studies on supervised learning-based LCZ classification fall into two categories: studies using classical shallow classifiers such as support vector machines, random forests, and canonical correlation forests (dos Anjos et al., 2017, Xu et al., 2017, Qiu et al., 2018); and studies using solutions based on deep learning (Xu et al., 2017). The latter approach is inspired by the goal of improving LCZ classification through the use of abstract low- and high-level features learned from data and has shown significant potential for use in other tasks, including object detection, image recognition, and semantic segmentation (He et al., 2016, Zhu et al., 2017, Mou et al., 2017, Wang et al., 2018, Mou et al., 2019). However, LCZ classification accuracy is probably limited by a general disregard of temporal information, an important shortcoming given that more than half of the 17 LCZs are expected to change their appearances and spectral characteristics over the course of a year. Recently, the strong potential of recurrent neural networks (RNNs) to harness the (temporal) dependency contained in (multi-temporal) image sequences has been demonstrated through various applications, including crop identification (Rußwurm and Körner, 2017), speech recognition (Greff et al., 2017), multi-label aerial image classification (Hua et al., 2019), time series classification (Interdonato et al., 2019), and change detection (Lyu et al., 2016, Mou et al., 2019). However, it remains unknown how RNNs can improve LCZ classification accuracy using multi-spectral remote sensing images, especially over a large area.

Within this context, the primary goal of our study was to provide further insights into the methodological aspects of LCZ mapping from multi-seasonal earth observation data using deep learning based methods. This study is an extension of our previous work in which we applied a residual convolutional neural network (ResNet) without consideration of temporal information and published the following findings:

• Comparable LCZ classification accuracies can be achieved using Sentinel-2 or Landsat-8 imagery; auxiliary data, such as OpenStreetMap (OSM), Global Urban Footprint (GUF), and VIIRS nighttime light, is not really necessary (Qiu et al., 2018).
• As a result of inter-class similarity and the class imbalance problem in reference dataset, it is difficult to differentiate building heights and distinguish similar LCZs using Sentinel-2 images alone (Qiu et al., 2018, Qiu et al., 2018). For example, LCZ 2 (*Compact mid-rise*) tends to be falsely classified into LCZ 3 (*Compact low-rise*) and LCZ 1 (*Compact high-rise*).
• Despite of a relatively low LCZ mapping accuracy (with average accuracy of about 50%), a quite high weighted accuracy (95%) is achievable (Qiu et al., 2018). As weighted accuracy focuses more on distinguishability between the urban and natural classes than on distinguishability between similar LCZs (such as *Compact high-rise* and *Compact mid-rise*), a high weighted accuracy indicates that the urban super-class can be accurately distinguished from the natural classes.

Based on our previous results, we were motivated to derive a new, simplified land cover classification scheme containing 6 aggregated classes. The correspondence between these derived urban land cover classes and the original LCZs is shown in Table 1. Based on the high weighted accuracies achieved in our previous work (Qiu et al., 2018), we believe that it is easier to classify these six urban land cover classes even if only optical satellite images are used. In a subsequent step, the classification results can be used, for instance, as a basis for further complete LCZ classification and human settlement extent and type classification.

**Table 1:** Derived land cover classes and their correspondence to original LCZs.

Label	Semantic class	LCZ
1	Compact built-up area	1, 2, 3
2	Open built-up area	4, 5, 6
3	Sparsely built	9
4	Large Low-rise, Heavy industry	8,10
5	Vegetation	A, B, C, D
6	Water	G

In this study, we focused solely on the freely available Sentinel-2 images (Radoux et al., 2016), which provide the potential for continental-scale or even global mapping. Our study was primarily intended to mainly provide answers to the following questions:

• Can land cover classification benefit from the temporal information contained in multi-seasonal Sentinel-2 images?
• How can an appropriate network architecture capable of extracting spectral-spatial-temporal features be designed to improve the classification accuracy?
• How can each LCZ benefit from the temporal information contained in multi-seasonal images?

To answer these questions, we investigated two approaches to exploiting the multi-temporal information within multi-seasonal Sentinel-2 images: using state-of-the-art convolutional networks architectures, i.e., ResNet; and using long short-term memory (LSTM). The temporal dependency can be modeled by either simply stacking multi-seasonal images or using a recurrent network, using an assumption under both modelling approaches that the spectral information for different land cover classes exhibits different patterns that change by season, which is obviously true for land cover classes such as *Dense trees* and *Low plants* (Rußwurm and Körner, 2017).

The remainder of this paper is structured as follows. In Section 2, we describe the proposed network architecture for classification. Section 3 provides detailed descriptions of the study area, multi-seasonal Sentinel-2 images, experimental setup, and reference ground truth data and pre-processing methods followed by an illustration of comparative classification accuracy and resulting land cover and LCZ maps. In Section 4, we answer our research questions based on the interpretation and analysis of experimental results and discuss remaining challenges and their possible solutions in future work. Finally, Section 5 summarizes and concludes the work.

## 2 A recurrent residual network architecture for land cover classification

When using multi-seasonal Sentinel-2 images as inputs for deep learning-based land cover classification, two approaches can be used to exploit temporal information. The first is to stack all images from different seasons as inputs of networks. The other is to treat multi-seasonal images as a changing representation of urban environments and to adapt state-of-the-art recurrent networks to model temporal dependencies of those images.

**Fig. 2:**
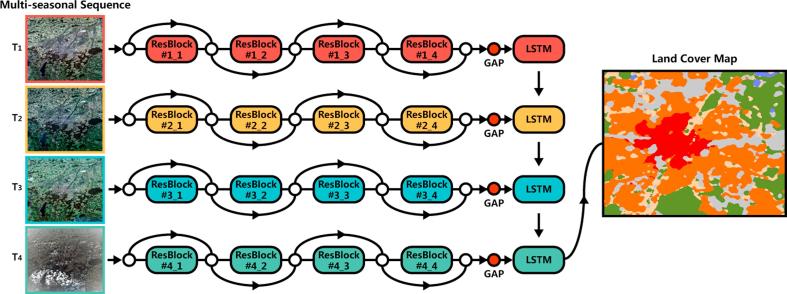
Re-ResNet network architecture for urban land cover classification. Here, $T_{1},T_{2},T_{3},T_{4}$ are the four seasons.

The proposed recurrent residual network architecture (Re-ResNet) is illustrated in Fig. 2. Under the Re-ResNet approach, a ResNet sub-network is applied to learn spectral-spatial features reflecting the multi-spectral data of individual seasons and an LSTM sub-network is used to model four-season temporal dependencies. The ResNet and LSTM sub-network are integrated into the Re-ResNet. Notably, these two components are integrated seamlessly into an end-to-end trainable network architecture based on the fact that both of them are fully differentiable. This enables the complementary information within discriminative spectral-spatial and temporal features to be learned for urban land cover classification. The LSTM is integrated with the ResNet in the manner shown in Fig. 2. In the network, extracted spatial features by ResNet are sequentially input into LSTM. In this manner, memory cells can be exploited to maintain temporal information from which temporal dependencies can be learned for land cover classification. The ResNet and LSTM sub-networks will be further described in the following sections.

### 2.1 Spectral-spatial feature extraction via the ResNet sub-network

**Fig. 3:**
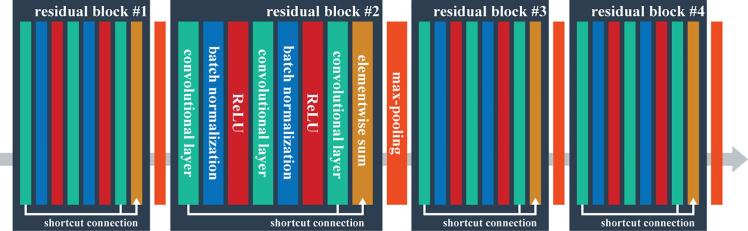
Architecture employed by ResNet sub-network. The input is an image patch and the output is either the learned spatial-spectral features (if used with the LSTM sub-network) or class labels (if directly used for classification).

We use a ResNet in the proposed method because such structures have demonstrated their ability to outperform others in image recognition on several benchmark datasets, including the Street View House Numbers (SVHN) and ImageNet datasets (He et al., 2016). The specific ResNet architecture adapted for our study is shown in Fig. 3. The adaptations are primarily implemented as a work-around of the 32×32×10 input patch size, which makes it impossible to directly employ original or pre-trained networks. Here, the filter size of the first convolutional layer of the original ResNet has been changed from 7×7 to 3×3 to maintain the spatial information within the input image patch.

The ResNet has a total of four residual blocks, each comprising three convolutional layers and one shortcut for bypassing two successive convolutional layers. Through the shortcut connection, the output of two stacked convolutional layers are added to the output of the first of the three convolutional layers. The convolutional layers have a rather small (3×3 pixel) receptive field with a stride of one pixel. The number of feature maps increases when the blocks become deeper, with a doubling occurring after every block. After each block, max-pooling is performed with a stride of two pixels.

### 2.2 Temporal feature extraction via the LSTM sub-network

An RNN is a type of neural network in which the conventional feedforward neural network architecture is extended with loop-containing connections. Unlike feedforward networks, RNNs are good at dealing with dependent and sequential input data with recurrent hidden states, the activation of which at each time step is dependent on the results of previous time steps. As a result, the network is able to exhibit a dynamic temporal behavior that is very similar to our goal of modeling temporal changes in land cover over different seasons. There are three primary types of RNNs: fully connected RNNs, LSTM networks, and gated recurrent units (GRUs). In this study, an LSTM architecture was used to construct a recurrent sub-network in our Re-ResNet because such networks have proven to be a quite powerful tool for modeling temporal concepts (Donahue et al., 2015).

**Fig. 4:**
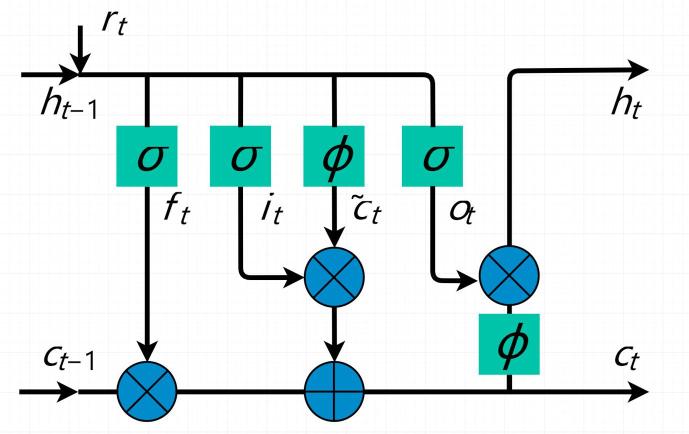
LSTM unit used in the Recurrent sub-network. Here, *t* indicates the current season and t-1 indicates the previous season; thus, t=1,2,3,4.

Fig. 4 shows the LSTM units employed in the Recurrent sub-network. Each LSTM unit employs three gates: an input gate $i_{t}$, a forget gate $f_{t}$, and an output gate $o_{t}$:

• The input gate $i_{t}$ adjusts the amount of new information ${\overset{∼}{c}}_{t}$ that will be additionally stored to the memory cell.
• The forget gate $f_{t}$ makes it possible for the memory cell to throw away already stored information. By summing the incoming information adjusted by the input gate $i_{t}$ with the previous memory adjusted by the forget gate $f_{t}$, the memory cell $c_{t-1}$ can be updated to $c_{t}$.
• The output gate $o_{t}$ enables the memory cell $c_{t}$ to exert an influence on the current hidden state $h_{t}$ before outputting $h_{t}$.

At season *t*, given an input $r_{t}$ (spatial features extracted by the ResNet sub-network), a hidden state $h_{t-1}$ from the previous network output, and the previous memory cell $c_{t-1}$, the LSTM unit is updated using the following equations:

(1) $$\left\{\begin{array}{c} i_{t}=\sigma (W_{\mathrm{ri}}r_{t}+W_{\mathrm{hi}}h_{t-1}+b_{i})\\ f_{t}=\sigma (W_{\mathrm{rf}}r_{t}+W_{\mathrm{hf}}h_{t-1}+b_{f})\\ o_{t}=\sigma (W_{\mathrm{ro}}r_{t}+W_{\mathrm{ho}}h_{t-1}+b_{o})\\ {\overset{∼}{c}}_{t}=\phi (W_{\mathrm{rc}}r_{t}+W_{\mathrm{hc}}h_{t-1}+b_{c})\\ c_{t}=f_{t}⊙c_{t-1}+i_{t}⊙{\overset{∼}{c}}_{t}\\ h_{t}=o_{t}⊙\phi (c_{t}) \end{array}\right)$$

in which the hyperbolic tangent function ϕ(a),

(2) $$\phi (a)=\frac{e^{a}-e^{-a}}{e^{a}+e^{-a}}$$

squashes the activations into the range [1, 1] and the sigmoid nonlinear function σ(a),

(3) $$\sigma (a)=\frac{1}{1+e^{-a}}$$

squashes the activations into [0, 1] to generate the three gates (Greff et al., 2017). The sets *W* and *b* are the weight and bias terms, respectively. ⊙ denotes element-wise multiplication involving computations with gates. The memory cells and gates have the same vector size. $h_{0}$ is initialized as 0. In our case, the output from the last season, $h_{4}$, is exploited for classification.

All of the networks are trained from scratch using the TensorFlow framework with a mini-batch size of 32. The Nesterov Adam (Sutskever et al., 2013) optimization algorithm is applied owing to its faster convergence relative to the standard stochastic gradient descent (SGD) with momentum algorithm. The Nesterov Adam parameters are fixed as recommended to $\beta_{1}=0.9,\beta_{2}=0.999$. The initial small learning rate of 0.0002 is divided by five at the error plateaus. Softmax is utilized as the activation functions for the final fully connected layer that outputs the class-wise probabilities for the various land cover classes.

## 3 Experimental results

### 3.1 Study area and cross validation setup

The study areas were seven cities locating in central Europe: Paris, Amsterdam, Cologne, Munich, Milan, London, and Berlin.

**Fig. 5:**
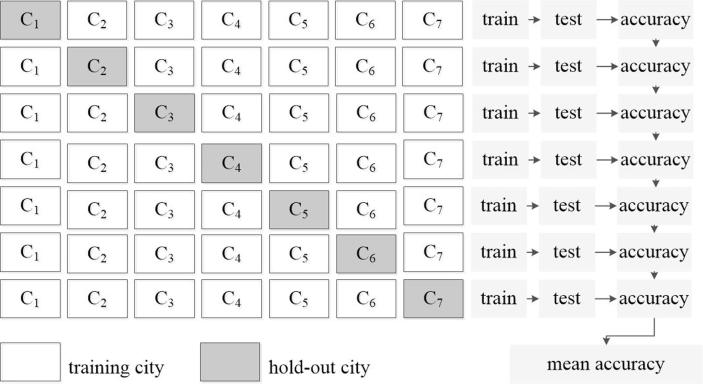
Cross validation experimental setup. The C_i denotes the city and each row indicates one experiment. In each experiment, the hold-out city was left for testing while the other cities were used for training.

To fully validate the proposed approach, we designed a cross-validation experimental setup, which is illustrated in Fig. 5. In total, seven test experiments were carried out: in each, one city was used for accuracy assessment while the other six were used for training networks. The classification accuracies for the individual test cities and the results averaged over all seven test cities (the mean accuracy shown in Fig. 5) were then summarized for further comparison and discussion.

### 3.2 Multi-seasonal Sentinel-2 images and LCZ reference data

**Fig. 6:**
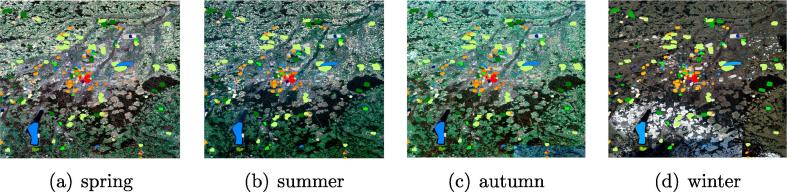
The exemplary multi-seasonal images over Munich, Germany. The colorful labels are the LCZ reference dataset.

For each city, we used Google Earth Engine (GEE) (Gorelick et al., 2017) to create four mostly cloud-free Sentinel-2 images covering each of the four seasons from winter 2016/2017 to autumn 2017. The multi-seasonal images of Munich, Germany are shown in Fig. 6 as examples. The multi-seasonal images are prepared using a cloud-based engineering approach that allowed us to aggregate mostly cloud-free images over rather concise time windows instead of the enter year. The approach relied on both pixel-wise cloud detection and the combination of multi-temporal information over comparably short time periods, an approach that represents a compromise between addressing the cloud problem, which is unavoidable when using optical satellite images, and retaining as much multi-temporal information as possible.

We used 10 of the 13 Sentinel-2 imagery bands: B2 (Blue), B3 (Green), B4 (Red), and B8 (Near-infrared) with a 10-m ground sampling distance (GSD); and B5 (Red Edge 1), B6 (Red Edge 2), B7 (Red Edge 3), B8a (Red Edge 4), B11 (Short-wavelength infrared 1), and B12 (Short-wavelength infrared 2) with a 20-m GSD, which were up-sampled to a 10-m GSD using cubic resampling. Bands B1 (Aerosols), B9 (Water vapor), and B10 (Cirrus) were not employed, as they mostly include information regarding the atmosphere and therefore have limited relevance to land cover differentiation. No additional extracted index measures or external auxiliary datasets were exploited, as our previous work revealed that they had limited contributions to deep learning-based methods (Qiu et al., 2018).

**Fig. 7:**
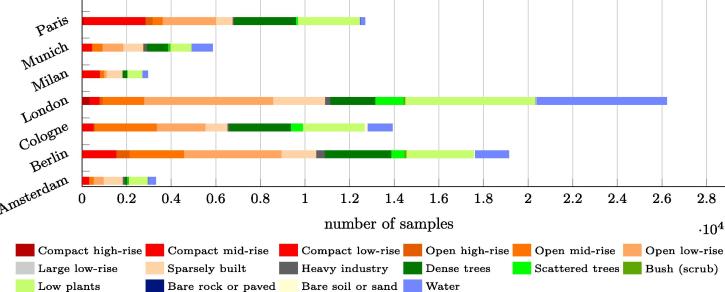
Number and distribution of LCZ samples in each of the seven test cases.

The LCZ reference labels for the study areas were taken from the LCZ42 dataset (Zhu et al., submitted for publication), which was used to provide ground truth data. The numbers of LCZ samples available for the seven cities are shown in Fig. 7. Only 16 of the 17 LCZs were considered in this study, as LCZ 7 (*Lightweight low-rise*), which primarily represents slums, isn’t existing in the study areas. Fig. 7 also reveals a class imbalance problem among the cities that might have hindered meaningful interpretation of experimental results. To address this, we performed a data augmentation procedure before combining the LCZs into land cover classes.

### 3.3 Data augmentation and accuracy assessment strategy

**Fig. 8:**
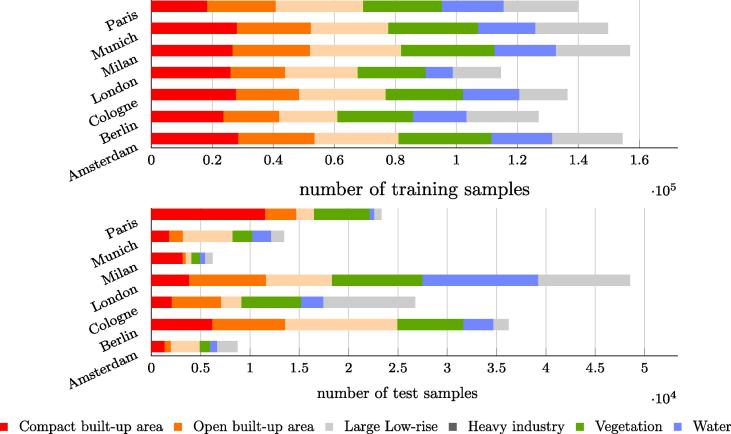
Number of training (top) and test (bottom) land cover samples used in each experiment. The training samples are balanced but the test samples are not balanced, which is taken into consideration during the accuracy assessment.

Data augmentation was carried out for LCZs 1, 3, 9, 10, 2, 8, and G by performing horizontal and vertical flip and rotation. In addition, class 3, i.e., LCZ9, was oversampled by using its samples repeatedly, to balance the land cover samples. Finally, the LCZ classes were combined by applying the scheme shown in Table 1. Fig. 8 shows the number of training and test samples for the seven experiments. The training samples were balanced for the six land cover classes, while the test samples were left unbalanced. To overcome the potential problems arising from the use of unbalanced samples in the test data, we applied the following strategy. For each experiment, 20 accuracy assessments, in which the same number of class samples were randomly chosen and assessed, were carried out. The number of chosen samples was bound by the minimum number of samples contained within each class. The averaged results of the 20 repetitions were then used for the final comparison and analysis.

As this process resulted in a completely balanced set of test samples, only two measures had to be used for accuracy assessment: overall accuracy (OA) and kappa coefficient.

### 3.4 Comparative accuracy of different approaches to extract multi-temporal information

The classification results obtained using the respective approaches are listed in Table 2, in which av-ResNet denotes the approach in which ResNet was used independently over all four seasons and the classification accuracies were averaged over the seasons and st-ResNet denotes the approach in which ResNet was applied with the seasonal images stacked as the network input. The classification accuracy of the proposed Re-ResNet was superior to those of both the av-ResNet and st-ResNet approaches. In addition, the accuracies differed significantly by season, with the spring and autumn results providing higher-accuracy results and the summer and winter results providing poorer-accuracy results. The st-ResNet approach was able to classify at an accuracy level between the highest and lowest levels for all four seasons and with an accuracy slightly lower than that of av-ResNet.

**Table 2:** Comparison of classification accuracy by approach based on results averaged over the seven test cases.

Temporal information	Approach	OA	Kappa
Not considered	Spring	82.7%	0.79
	Summer	81.2%	0.77
	Autumn	82.7%	0.79
	Winter	77.9%	0.74
	av-ResNet	81.1%	0.77
Considered	st-ResNet	79.8%	0.76
	Re-ResNet	84.0%	0.81

**Fig. 9:**
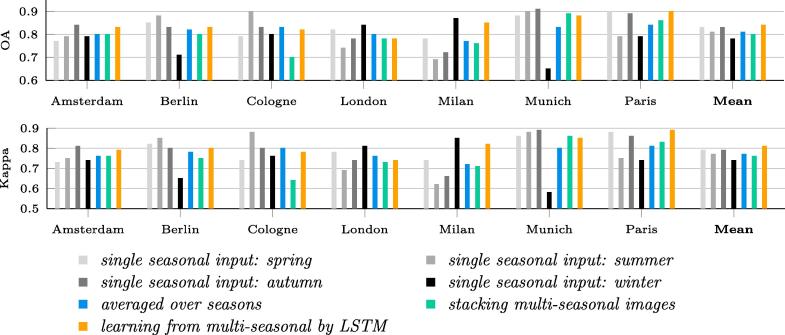
OA and Kappa resulting from different approaches. The training samples for each city are from the other six cities. The last column (mean) represents the averaged results over all seven test cases.

A detailed comparison of the applications of the respective approaches to the different test cases is shown in Fig. 9, in which the final column (Mean) corresponds to the data in Table 2. It is seen from the figure that the results mentioned above apply to most the cities. Furthermore, for most cities the highest degree of accuracy is obtained for single-season input, although the season producing the best results varies by city. For example, the summer input is most accurate for the Berlin and Cologne test cases but least accurate for the London and Milan cases. Overall, no single-season results are more accurate than those produced by Re-ResNet.

**Fig. 10:**
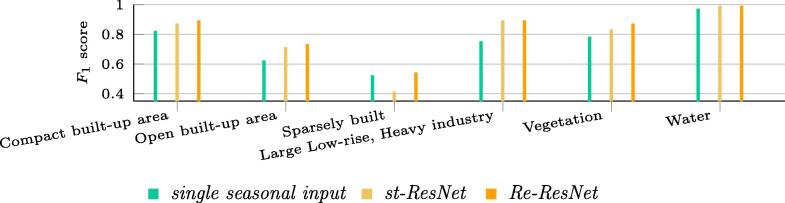
$F_{1}$ scores for land cover classes produced by different approaches.

The contributions of temporal information by the respective individual land cover classes are shown in Fig. 10.

### 3.5 Classification accuracy of Re-ResNet

**Fig. 11:**
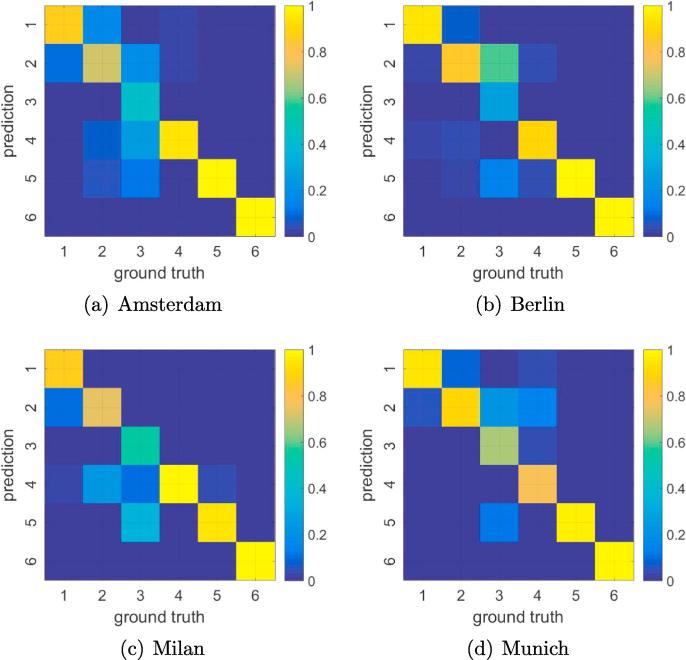
Confusion matrices for four sample experiments.

The class-wise classification accuracies of the Re-ResNet results for the seven experimental sets are listed in Table 3. The proposed approach is able to produce very highly accurate results for two classes (Vegetation and Water), highly accurate results for three classes (Compact built-up area, Open built-up area, and Large low-rise and heavy industry), and results with relatively low accuracy for the Sparsely built class. These findings are reflected in the corresponding confusion matrices shown in Fig. 11, in which class 3 (*Sparsely built*) is often misclassified as class 5 (*Vegetation*), class 2 (*Open built-up area*), and class 4 (*Large low-rise and heavy industry*).

**Table 3:** Class-wise classification accuracies of the Re-ResNet approach for the seven test cases.

	Class	Amster.	Berlin	Cologne	London	Milan	Munich	Paris	Mean
Recall (%)	1	87	95	80	78	88	94	90	88
	2	71	86	66	94	75	90	85	81
	3	43	27	74	8	54	66	75	50
	4	95	91	73	89	100	77	98	89
	5	99	100	99	98	94	98	98	98
	6	100	100	100	100	98	100	96	99
Precision (%)	1	81	93	83	95	99	87	95	91
	2	70	57	76	59	90	67	92	73
	3	99	95	69	82	96	93	95	90
	4	73	93	95	90	70	99	86	87
	5	85	83	76	67	72	89	80	79
	6	98	99	97	99	100	100	100	99

### 3.6 Classification maps of Re-ResNet

**Fig. 12:**
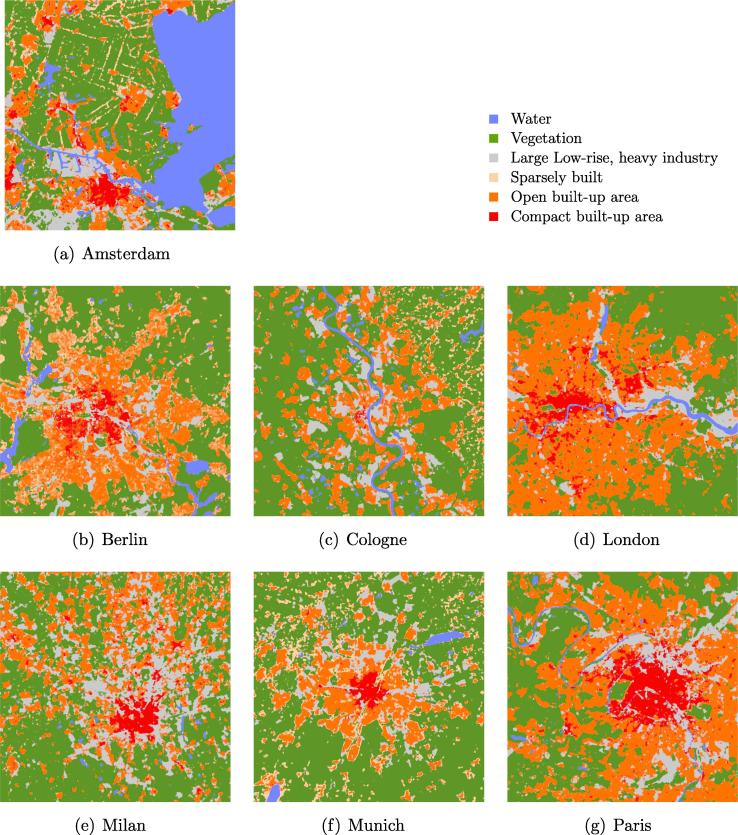
Land cover maps produced for the seven cities. To present an equivalent size for all test cases, only a part of each city map, covering about $39.7\times 39.7\;{\text{km}}^{2}$, is shown. The GSD of each map is 50 m.

**Fig. 13:**
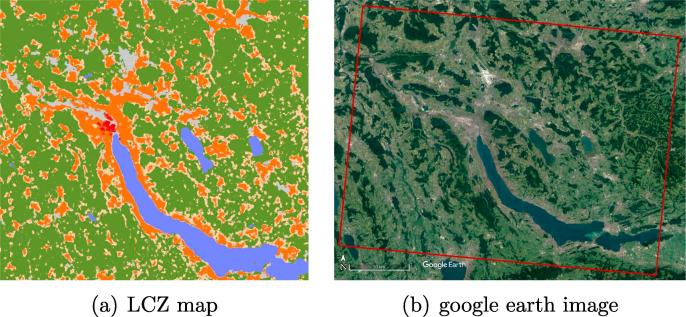
Land cover map of Zurich, Switzerland, from which no training samples were used for any of the experiments in the study. The GSD of the map is 50 m. The corresponding Google Earth image is shown for comparison.

Fig. 12 shows sample classification maps produced for the seven cities using the trained Re-ResNet with the land cover samples shown in Fig. 8. An additional land cover map of Zurich, Switzerland is shown in Fig. 13. Note that no training samples from Zurich were used for training the network. All of the maps display reasonable urban structures without obvious noise.

**Fig. 14:**
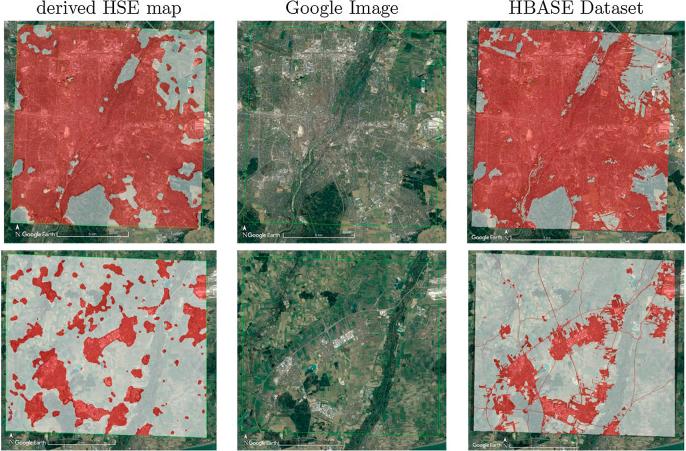
(L-R): Derived land cover maps, optical images taken from Google Earth, and the HBASE dataset-derived maps of the city center (top) and suburbs (bottom) of Munich, Germany. The GSD of the land cover maps is 10 m. The red areas in the land cover maps are combined from class 1, 2, 3, and 4 in Table 1, indicating the human settlement extent (HSE). The red areas in the HBASE dataset maps indicate built-up areas.including roads. (For interpretation of the references to colour in this figure legend, the reader is referred to the web version of this article.)

A zoomed-in view of the city center and a suburban area of Munich, Germany are shown in Fig. 14. The corresponding optical images taken from Google Earth and the Global Human Built-up And Settlement Extent (HBASE) Dataset (Wang et al., 2017) are shown for comparison. There is a general consistency between the two sets of images in terms of urban extent, particularly in the city center area. Furthermore, the proposed approach is able to map several small villages in the suburban area that are missing from the HBASE dataset.

**Fig. 15:**
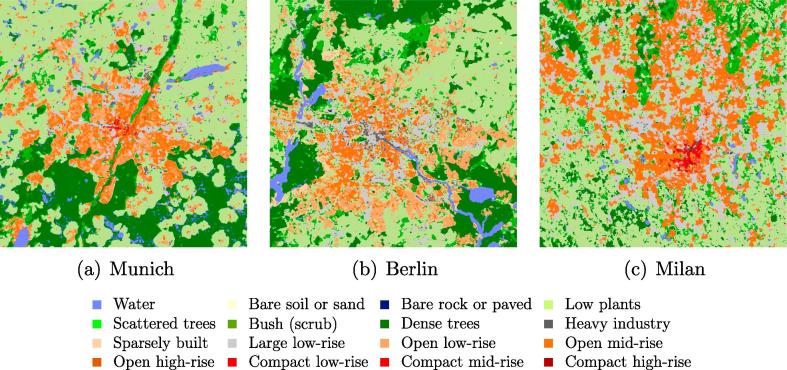
LCZ maps of three cities. To present a comparative size for the respective test cases, only a $39\times 39\;{\text{km}}^{2}$ section of each map is shown.

To illustrate the potential of Re-ResNet for large-scale LCZ mapping using a given number of reference LCZ samples, LCZ maps of three sample cities Munich, Berlin, and Milan are shown in Fig. 15. The three maps were produced using three networks that were trained with the original LCZ samples that did not represent the target cities. For example, no training samples from Munich were used for training the network to produce the LCZ map of Munich.

### 3.7 Improvement achieved using Re-ResNet in terms of number of training samples

**Fig. 16:**
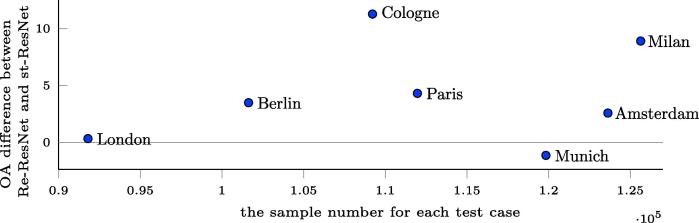
OA difference between Re-ResNet and st-ResNet, as a function of number of training samples. A negative OA difference indicates that Re-ResNet does not provide an improvement over st-ResNet. The values used in this figure are the same as those reported in Section 3.

To illustrate teh relative advantage achieved by using Re-ResNet over st_ResNet instead of st-ResNet, we graph the difference between the Re-ResNet and st-ResNet OA scores against number of training samples in Fig. 16.

**Fig. 17:**
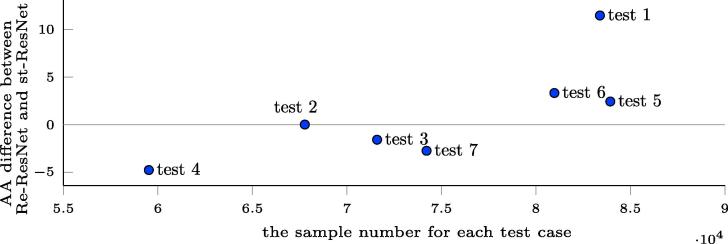
AA difference between Re-ResNet and st-ResNet, as a function of number of training samples. A negative value means that Re-ResNet does not provide improvement over st-ResNet. The values are taken from the LCZ classification, not the land cover classification developed and described in Section 3. AA is used to address the class im.balance problem.

We then carried out seven additional LCZ classification experiments in which st-ResNet and Re-ResNet were applied using different numbers of training samples. The improvement in averaged accuracy (AA) obtained by using Re-ResNet instead of st-ResNet is apparent in Fig. 17.

## 4 Discussion

Both the classification accuracy and the produced land cover and LCZ maps in Section 3 indicate that the proposed network architecture and classification procedure are promising. The results also suggest that multi-seasonal Sentinel-2 images should be employed for operational production at a similar scale. It should be mentioned that the resulting classification maps are not ready to be directly used for detailed climate-related studies: Whereas the original 17 LCZ classes were designed bearing climate-relevant surface properties in mind (Bechtel et al., 2015), the simplified class scheme utilized in this study exhibits class-internal heterogeneities with respect to climate-relevant parameters. For instance, there is a significant difference in the mean night-time air temperature between LCZ class 5 and 6, even though the highest difference appears between LCZ class 2 and D (Fenner et al., 2017). Nevertheless, our LCZ-derived land cover maps can well be used for preliminary or coarse urban climate-related studies, as they provide the basic two-dimensional information about urban morphology (i.e. whether a neighborhood can be characterized as compact or as open built-up area) and also indicate industrial zones where usually the highest land surface temperature appears (Huang and Wang, 2019). Additionally, using the simplified land cover classification results as basis, a complete LCZ classification can be achieved by adding multi-sensor and multi-temporal information, such as that provided by LiDAR and satellite images acquired by other sensors (Xu et al., 2018). This way, comprehensive studies regarding urban heat islands and energy resilience can benefit from the mapping results.

In light of these experimental results, the contribution of temporal information to the LCZ-derived land cover and LCZ classification and the comparative advantage of the proposed network will be discussed in this Section. Furthermore, the remaining challenges and possible solutions will be analyzed.

### 4.1 The contribution of temporal information to land cover classification

A comparison of the classification accuracies resulting from approaches with- and without considering temporal information (in Table 2) reveals that land cover classification accuracy is improved through the use of the temporal information contained in multi-seasonal Sentinel-2 images, as both OA and Kappa are higher for the approaches that apply temporal information. It is seen from the table that even simply stacking multi-seasonal images and processing them with ResNet improve OA and Kappa from 77.9% to 79.8% and from 0.74 to 0.76, respectively, relative to a baseline ResNet with the winter-image as input. This suggests that multi-temporal information should definitely be considered for similar land cover mapping. This holds especially as we are living in the “golden era of Earth observation,” which is characterized by an abundance of sensors that regularly provide new remote sensing data. Additionally, attention should be put on the specific chosen time period considering land cover and land use change during the period, as we are currently living in a rapidly urbanizing world.

It is also seen from Table 2 and Fig. 9 that limited improvement is achieved by st-ResNet (stacked images as input). This is probably due to the varying qualities of multi-seasonal images. For example, as shown in Fig. 6 the images of Germany in winter tend to be affected by cloud or snow. Thus, incorrect predictions from cloudy images can lower resulting accuracies.

From Fig. 9, it can be seen that the accuracies for Munich in summer and Paris in spring are relatively high, while those for Milan in summer and autumn are lower. The transferability of trained classifiers depends on the similarities between the test and training cities, a result that was similarly reported in Demuzere et al. (2019). The differences among the cities under study include, but are not limited to, the following: climate, culture, and urban land cover structure. All of these aspects play a role in inter-city similarity. For instance, the relatively-low latitude of Milan leads to a slight shift in the seasonal characteristics, which probably contributed to the comparably low single-season accuracies achieved for autumn and summer. When additionally considering the urban ecoregions of the test cities (Schneider et al., 2010), Milan belongs to the “temperate mediterranean”, while the other cities belong to the “temperate forest in Europe”, which accounts for the lower transferred accuracies achieved for Milan. Furthermore, the seasonal images were created by a multi-temporal mosaicking process that was applied for the sake of cloud removal. As a result of the mosaicking, the intra-seasonal differences for some cities were not as big as for others. This may have led to cases in which, for example, the spring image for one city was dissimilar to those for the other cities. In summary, it was not possible to clearly identify whether any specific season dominated the others for all test cases. These findings indicate that single-season images were not able to fully exploit Sentinel-2 data for land cover mapping at similar scales. It also highlights the need for a sophisticated network to exploit temporal information more effectively.

### 4.2 The contribution of Re-ResNet to the extraction of temporal information

The effect of the proposed network architecture is apparent from the improvement achieved relative to st-ResNet in terms of OA and Kappa, as indicated in Table 2. The Re-ResNet result accuracies are the highest among all of the approaches. Because the same number of samples was used for training each network, this improvement can be attributed only to the proposed Re-ResNet network, which learns temporal information via its Recurrent sub-network and spatial and spectral features from the baseline ResNet. Given its more extensive load of trainable parameters, it is not surprising that Re-ResNet is better at this classification task. However, as shown in Fig. 9, Re-ResNet did not provide the best accuracy for all test cases. One reason is that Re-ResNet requires a high amount of training data because its architecture contains more trainable parameters.

Fig. 16 provides some evidence to the possible causes, based on which, it can be speculated that the number of training samples plays a role in the achievable improvement. However, the number of training samples is quite close across the seven test cases, as a result of the data augmentation applied in the data pre-processing period. Better evidence for the effect of the number of training samples is found in Fig. 17, in which Re-ResNet provides an improvement over st-ResNet only in the three test cases with the three highest number of training samples. As the amount of training data increases, the advantages of Re-ResNet with respect to both land cover and LCZ classification become increasingly apparent.

Nevertheless, the accuracy of the Re-ResNet can be lower than that obtained from a single-season input, as is true in the case of London and Munich. This occurs as a result of the varying qualities of multi-seasonal images, as shown in Fig. 6. However, over all seven test cases, no single season provides more accurate results than Re-ResNet. These observations suggest a potential avenue for future work of selecting seasons with higher image qualities prior to classification. To this end, techniques for cloud removal should be thoroughly investigated.

### 4.3 The contribution of temporal information for different land cover classes

Fig. 10 shows that Re-ResNet did not provide better results than st-ResNet for all six classes. We expected to observe the improvements for class 2 (*Open built-up area*), 3 (*Sparsely built*), and 4 (*Vegetation*), as these show clear changes over different seasons, but the improvements seen for *Heavy industry*, *Compact built-up area*, and *Water* motivate us to further investigate the learned features of Re-ResNet in future research.

In addition, as seen in Table 3, class 3 (*Sparsely built*) was not accurately mapped by any of the approaches, including Re-ResNet. One reason is that this class can be easily and understandably misclassified as *Open built-up area* and *Vegetation*. Its accuracy can be potentially improved by adding more samples of class 3 to the training data or by resorting to complementary datasets. In addition, it is possible that some Sparsely built training samples actually contain no buildings, i.e., that they are noisy samples. This motivates us to try to develop robust approaches capable of dealing with label noise in an appropriate manner.

## 5 Conclusion

The goal of this study was to contribute to the automatic mapping of large-scale LCZ-derived land cover with a given amount of reference data. In particular, we investigated transferability among different cities. For this, we proposed a novel recurrent residual network architecture, called Re-ResNet, that combines a residual convolutional neural network with a recurrent neural network. Re-ResNet is able to learn a joint spectral-spatial-temporal feature representation in a unified framework using multi-seasonal Sentinel-2 imagery as the network input. The method was validated over a large-scale study area spreading over seven central European cities, with the experimental results demonstrating that the proposed approach outperformed ResNet with single-seasonal images as inputs and st-ResNet with stacked multi-seasonal images as inputs. Systematic analysis based on the results also clearly revealed the effectiveness of using the temporal information contained in multi-seasonal, multi-spectral Sentinel-2 imagery for urban land cover classification.

Even though we were able to demonstrate that the proposed Re-ResNet can generally improve classification accuracy, we anticipate a need for two further avenues of investigation and research: differentiating the Sparsely built class from the Open built-up area and Vegetation classes, and an application-driven analysis of the resulting maps. After solving these two problems, it should be possible to produce land cover and urban human settlement maps with even higher accuracy to provide more reliable morphological information on cities worldwide that can be used to obtain further knowledge and insight into the ongoing process of urbanization.
